# Relative Wellbeing of Women Maltreated as Children

**DOI:** 10.1177/10778012211058218

**Published:** 2021-12-13

**Authors:** Linda Arnell, Åsa Källström, Hrafnhildur Gunnarsdottir

**Affiliations:** 1 School of Law, Psychology and Social Work, 6233 Örebro University, Örebro, Sweden; 2 School of Public Health and Community Medicine, Institute of Medicine, 3570 University of Gothenburg, Gothenburg, Sweden; 3 Department of Health Sciences, University West, Trollhättan, Sweden

**Keywords:** childhood maltreatment, qualitative, Sweden, wellbeing, women

## Abstract

This study explores and analyzes how adult women in Sweden exposed to childhood maltreatment describe wellbeing, by using a thematic analysis of 22 semi-structured interviews with women maltreated as children. The results show that wellbeing was described as relative to both social norms and the childhood experiences and constituted four dimensions: Material and/or economic; Social and relational;Emotional; and Physical and/or mental. This study concludes that it is important to consider the relative and multiple ways wellbeing can be experienced and understood and to problematize norms of wellbeing, acknowledging the various ways people appraise their lives.

## Introduction

In addition to threatening a child's wellbeing, maltreatment during childhood can negatively affect one's adult life. Maltreatment—that is, neglect or physical, sexual, or emotional abuse—increases the risk of child mortality and morbidities and may havelasting effects on mental health (e.g., anxiety, depression, and posttraumatic stress symptoms), physical health (e.g., overweight, drug and alcohol misuse, and risky sexual behavior), violent and other criminal behavior, school problems, and social problems (e.g., Gilbert et al., 2009; Vahl et al., 2016). Similarly, childhood exposure to intimate partner violence entails a risk for developing emotional and behavioral problems and for greater exposure to other adversities (Holt et al., 2008; McTavish et al., 2016). Currie and Spatz Widom (2010) found that adults with documented histories of childhood maltreatment have lower levels of education, employment, and earnings and fewer assets as adults, compared to other adults. These outcomes are described as large with enduring consequences for maltreated children's economic wellbeing as adults (Currie & Spatz Widom, 2010).

However, not all victims of maltreatment in childhood experience negative consequences to the same extent. Some victims adapt positively despite severe adversity (Rutter, 2012; Wright et al., 2013). Approximately 15%–47% of those who experienced childhood maltreatment adapt positively in terms of social functioning and mental health and exhibit resilience (Domhardt et al., 2015). However, previous research on positive adaption (i.e., resilience) has mainly focused on conditions in childhood and paid little attention to adulthood conditions and experiences (Liebenberg & Moore, 2018).

Understanding what wellbeing means to adults subjected to maltreatment in childhood could provide insight into how to best support them. Given that women more often than men report having been maltreated as children and being negatively affected by it (Currie & Spatz Widom, 2010), studies of how victimized women experience, define, and describe wellbeing are particularly important. Against this background, this study explores and analyzes how adult women in Sweden exposed to childhood maltreatment describe wellbeing.

### Theoretical Approaches to Wellbeing as Objective and Subjective

The literature reveals two approaches to the conceptualization of wellbeing: the objective and the subjective. The objective approach focuses on measurable indicators such as material resources (e.g., income and housing) and social aspects (e.g., education and social networks) (Diener & Suh, 1997; Western & Tomaszewski, 2016). That is, the objective approach relies on external criteria for a “good life,” desirable qualities based on a value framework of an observer rather than the individual (Diener, 1984). The subjective approach, on the other hand, embraces how individuals themselves experience and appraise their lives, the events happening to them, their physical and mental health, and their living conditions, all in relation to their own standards (Diener, 2006; Diener & Suh, 1997). Subjective wellbeing includes both a cognitive evaluation of life circumstances (i.e., life satisfaction) and a hedonic value of life events and activities (i.e., happiness) (Diener, 2006; Kahneman et al., 2010). However, the subjective wellbeing that is associated with life satisfaction is not necessarily associated with happiness. For example, Kahneman et al. (2010) found that women in the United States as well as in France experienced a more satisfied life if they had a mate, had a good household income, had higher education, were employed, had children, and had good health and a trim figure, but none of these conditions necessarily contributed to their happiness.

Although the structure of wellbeing has been found to be similar across cultures, the content of wellbeing—that is, the specific sources from which individuals draw wellbeing—seems to be related to the subjective experience of time, enjoyable activities, and positive mood (Kahneman et al., 2010; Krueger et al., 2009). How individuals describe wellbeing has also been found to differ by culture, reflecting differing norms and social arrangements (Kahneman et al., 2010). For example, the specific sources from which the women in the United States and France draw wellbeing were slightly different, relating, for example, to the amount of time spent on childcare and the enjoyment derived from childcare (Kahneman et al., 2010). Furthermore, research shows that religion is associated more with wellbeing in countries where religion is of greater significance than in countries where it is not (Diener et al., 2011). Also, the experience of being connected to nature and subjective wellbeing are related, but differ from childhood experiences and culture (Capaldi et al., 2014). In addition, in some parts of the world subjective wellbeing, in terms of happiness, is related to good luck and fortune. In other parts of the world, good luck and fortune are considered something to be earned and deserved (Diener et al., 2018).

If, how, and how much major life events affect subjective wellbeing is, however, an issue of debate. Originally a paradigm of set-point, adaption level, or similar theories dominated the field (Headey, 2010). These theories state that individuals have a firm set-point or adaption level for their subjective wellbeing based on inherent personality traits. Accordingly, the major life events—for example, loss of a loved one, unemployment, divorce—can lead to a temporary decline in subjective wellbeing but after a while, the individuals will bounce back to their set-point or adaption level (Diener et al., 2018; Headey, 2010; Suh et al., 1996). This paradigm has been challenged by research finding that major life events can cause long-term changes in subjective wellbeing, either positive or negative (Headey, 2010) and that experiences of wellbeing most often are influenced by environment and circumstances (Diener et al., 2018), even though evidence also supports that personality is of importance for the scope of such changes as well as the direction (Diener et al., 2018; Headey, 2010).

Furthermore, it has been highlighted that individuals experience wellbeing and self-worth by comparing their situation with others—social comparison—especially people in a worse situation than themselves, or by comparing with a former self–temporal comparison—perceiving themselves as strong and capable to deal with an extreme situation and accordingly perceiving themselves and their lives in a more benevolent way (Ben-Zur, 2016; Janoff-Bulman, 1992). The significance of traumatic life events that occur during childhood for the theoretical understanding of wellbeing is less prominent, even though it is well established that childhood adversities—for example, maltreatment—are linked to poor wellbeing. It is also known that maltreatment in childhood does influence the developmental trajectories of personality and behavioral functioning (Kim et al., 2009).

Considering the understanding of wellbeing depending on the environment and circumstances as well as cultural context, it is important to study how individuals with different life-changing or life-shaping experiences describe wellbeing. Drawing on the research described above, knowledge on how women maltreated as children describe wellbeing seems particularly important.

## Method

This qualitative study explores and analyses, as previously noted, how women maltreated as children describe wellbeing. The definitions of childhood maltreatment as well as of wellbeing and functioning in adulthood were left to the interviewed women. Further, it was a subject of discussion with each woman before the interview to establish whether she defined herself as maltreated as a child and experiencing wellbeing and well-functioning as an adult and thus could participate in the project.

### Participants

Women who experienced childhood maltreatment were recruited through an announcement posted for two weeks in the news feed on Facebook among women aged 30–65 years living in the middle and western regions of Sweden (see @AWAREstudyGU on Facebook). Women were invited to participate if they had witnessed domestic violence or experienced any kind of physical, emotional, or sexual abuse and/or neglect from a close adult person during their childhood but considered themselves as having positive wellbeing and a well-functioning life as an adult. The women who were interested in participating submitted a statement of interest on a contact form on the project's website. These women were then contacted by the interviewers (second and third authors) through e-mail or telephone. During this contact, the women were provided detailed information about the study and made an appointment for an interview. Two women decided not to participate after the primary contact since, after further consideration, they did not define themselves as experiencing wellbeing. Inclusion of more participants to the study continued until we judged that each new interview did not add enough new information to what we already had to motivate the effort it demanded from participants and us.

The study included 22 women aged 31–64 years (mean age 48 years) living in Sweden. They had all experienced maltreatment by a close adult person as children:13 in the form of physical abuse by their biological, step- or foster father, 11 in the form of sexual abuse by their father and/or other relative, nine in the form of witnessing their father's severe violence against their mother, and 19 in the form of neglect. Thus, the vast majority had been repeatedly maltreated by a close adult person (most often a parent or stepparent) in multiple ways during a substantial part of their childhood; no one described only occasional events of maltreatment.

### Data Collection

Between March and May 2018, the second and third authors conducted 11 interviews each. Each interview lasted between 47 and 110 min and was audiotaped and transcribed verbatim. The interviews were conducted in a place chosen by the women, most often in a separate room at the University of Gothenburg or at Örebro University, but in some cases, the woman chose to be interviewed in a separate space at her home or at a café. Although this meant that other people could hear parts of these interviews, those women did not seem to mind and instead talked about their experiences matters-of-factly. The interviews were constructed around three themes: the women were asked to describe their own wellbeing and well-functioning as adults and why they considered themselves to be suitable for participating in the study; the women were asked to describe their childhood experiences; and the women were asked to describe their life journey from the childhood experiences to their present state of wellbeing. Follow-up questions were asked if needed, but the main focus was on the women's spontaneous responses to the questions—that is, the interviewers kept the focus on the women's stories.

### Ethics

Ethical approval was attained from the Regional Board of Ethical Vetting at Gothenburg (dnr: 258-17). The women received a written information letter sent by email immediately after being assigned to the study. The information was repeated verbally at the interview, including an assurance that the women could withdraw from the study at any time. In addition, the women were allowed to ask questions before they provided their signed informed consent and received information about where to seek help if the interview induced any difficult feelings. Pseudonyms have been used to protect the identities of the participants.

Several ethical issues are particularly important to acknowledge in research about maltreated women, as the sensitivity of the research sharpens ethical dilemmas and may reveal the limits of existing ethical theories (Fontes, 2004). First, women who participate in research interviews about their victimization may be speaking out in a context of disbelief, fear, and shame (Fontes, 2004). Therefore, in all contact with potential participants in the study, we pinpointed that the interviews would be conducted on the women's terms and that they were the bearers of the truth. Indeed, most of the women expressed that they appreciated the research focus on their narratives as survivors rather than victims and their experiences of wellbeing rather than negative consequences. Second, researchers must acknowledge that prior trauma may limit potential participants’ ability to understand the risks and benefits of participating in research and impair their judgment of whether participation is appropriate for them (Fontes, 2004). Although the women contacted us about participation after defining themselves as experiencing wellbeing and well-functioning as an adult, we utilized the principle of “ongoing consent” to deal with this. That is, sensitivity and responsivity to any negative reaction to the research situation in each contact and situation on a minute-by-minute basis throughout the interviews (cf. Cater & Överlien 2014). Third, researchers’ ambition to maximize the quality of data by using their empathic skills to help participants speak freely may risk that interviewees confuse the researcher's role and possibilities with a counsellor's and disclose information they never meant to disclose (Fontes, 2004). We addressed this by keeping this risk in mind during the interviews and by prioritizing protecting the identity of informants over revealing details in the results.

### Data Analysis

We used an inductive approach—that is, the women's narratives from the interviews guided the analysis. The interviews were analyzed using thematic analysis (Bryman, 2016). First, all authors read the interview transcripts from beginning to end to obtain an overall impression of the narratives in relation to the research question (Creswell, 2014). Next, the first author searched for a general pattern describing wellbeing, a strategy that showed that economic, social, and subjective assessments were seen as the main aspects of wellbeing. This general understanding of the narratives was then discussed among the authors while they reflected on the initial stage of the analysis. Finally, the first author closely reread the narratives specifically searching for different definitions and units of meaning (codes). This close reading produced four themes: material and/or economic dimensions of wellbeing; social and relational dimensions of wellbeing; emotional dimensions of wellbeing; and physical and/or mental dimensions of wellbeing. These units of meaning provide the basis for the theoretical understanding of the narratives. Therefore, wellbeing was described as constituting four dimensions, revealing a relative wellbeing. Thereafter, the first author sorted the empirical material in relation to each theme. The various codes and the four themes were illustrated by means of a mind map to make visible and illustrate the analysis. This mind map was discussed and evaluated jointly by all authors. When writing the results, each quotation has been reread in an attempt to refine the analysis and capture the entire content of the interviews, including similarities and differences in the narratives. The analysis was conducted on the Swedish interview transcripts and the excerpts were later translated into English.

## Results

The main finding of this study is that the women described wellbeing as relative. In addition, they described wellbeing as four interconnected dimensions: Material and/or economic; Social and relational; Emotional; and Physical and/or mental. However, these dimensions were not described in a vacuum as they were explicitly or implicitly related to either a set of social norms and/or their childhood maltreatment. Specifically, while material and/or economic and social and relational dimensions were described as relative to both social norms concerning what it means to be a well-functioning adult and citizen and their childhood maltreatment, emotional and physical and/or mental dimensions were primarily described as relative to what they had experienced as children. Below, we show how these dimensions constitute the women's descriptions of wellbeing as relative.

### Material and/or Economic Dimensions of Wellbeing Relating to Norms of What it Means to Be a Good Citizen

One important aspect of wellbeing in the women's descriptions was having access to material resources such as having an apartment or house and perhaps a summer house or a car, as well the means for obtaining these resources such as having a full-time job, a steady income, and possibly an education. Wellbeing defined in terms of such material and/or economic aspects was often explicitly or implicitly related to various social norms or expectations. The women related and (re)negotiated these norms in their narratives when describing their own wellbeing and everyday lives, a perspective exemplified in the following quotation:

I still think that I have managed to do well in life. I have an education, I have studied at the university, I have never been unemployed, I have a family, children. So, I’ve got things like this that are still quite important to function in society and I often have. […] Thus, I have always functioned in society. I have never been criminal or ended up in any kind of abuse of any kind, but I have had and still suffer from mental illness; that's how it is, and I struggle very hard with it. But despite that, I still think that I have attained a well-functioning life and […] I’m kind of highly paid, if it can be measured as wellbeing, but it's still […] that is, I have somehow succeeded with something in some way, managed. It has been a battle in some ways, but I have managed it anyway. (Diana)

Like Diana, the other women related their wellbeing to a well-functioning life, which was described in terms of having a good job and sometimes an education as well as a good financial and living situation. Although Diana highlights her job, children, and education to exemplify what well-being means for her, she also talks about a struggle with mental illness and also compares her situation with abuse and criminality. Describing having suffered from mental illness and avoided criminality by battling her way through life illustrates the contrast between norms and what the experience of maltreatment has meant for her. Like Diana pointing to her struggle and deviations from the norm, the normative idea of a well-functioning life was often specifically negotiated in relation to the harm the maltreatment had caused:

I’m not fully functional in society. And I will never be. I am injured both physically and mentally. And I will never fit in a small box the way society wants us to be. And somehow, I’m a little proud of that too. It feels good, the knowledge that there are different kinds of people, it's good. But I will never be able to function as an “ordinary person,” in quotation marks. And I’m very aware of that. I cannot […] for example, I have physical problems. I cannot work 100% like other 40-year-olds. And […] yes, I always have to be in control of my emotions, because I cannot completely relax, which means that I can't fully function in society. I cannot be like everyone else. […] I will never be able to live like as everyone else. I cannot. But I think I live a normal life. That's probably how I want to put it. I have a job. I drive a car. I have kids. I have a husband. We have a house [laughs]. I think I live a normal life outwardly. […] I’m functional out in the system [laughs], so to speak, even though I’m different. (Elisabeth)

Accepting limited possibilities and living the kind of life that many others strive for or a life that follows the normative ideas of adult life, Elisabeth describes a life she has chosen and adapted to her circumstances. The consequences and health issues related to trauma, following maltreatment in childhood, were often highlighted to describe why or how one's own life differs from the norm, but, as for Elisabeth and Diana, this did not impede them from defining their own life as well-functioning and as such renegotiating the normative idea of a “good life” and describing themselves as experiencing wellbeing. Accordingly, in the light of life situations, sometimes differing significantly from normative ideas of a “good life” and being a good citizen because of the harm of the childhood maltreatment, work, sometimes education, a home, and a secure financial situation were highlighted in the interviews as important for wellbeing.

### Social and Relational Dimensions of Wellbeing Related to Norms of What are Good Relations

Another important dimension of wellbeing in the interviews concerned relations, or more specifically good relations, characterized by, among other things, trust and support. Such aspects of life were generally given great importance in the women's narratives. Different relationships were described as fulfilling different needs and as having different functions in life and, therefore, various relationships were described as important for their wellbeing:

The most valuable are my relationships, my closest relationships, because without them I would probably feel very “naked” and very vulnerable. So that is my children and grandchildren and my husband, who has been with me for a while [35 years] [laughs]; they are the most important. (Frida)

Frida described wellbeing with reference to good relationships. Good relationships were described in terms of security, trust, and mutual respect as well as being free from all forms of violence. They were in various ways described as joyful parts of life, but they were also described as one's lifeline and what makes life worth fighting for. When describing the significance of good relationships, primary importance was given to children and a partner, but siblings, parents, friends, colleagues, and pets were also given great importance.

To highlight the importance of good relationships, many women made a distinction between good relationships in their present life and destructive relationships in their past life. This was mainly done by describing a childhood affected by violence, abuse, and/or neglect, but it was also done in relation to contemporary relationships with, for example, ex-partners. One example of this is found in Magdalena's narrative when she compares present and past relationships:

Magdalena: I’m not afraid of Robert, but he is offensive in his communication, the last time, I had such a pulse, I didn't dare to go outside the house because I thought he was standing somewhere. I had the same feeling with my dad. And I thought that wasn't possible because I’m not afraid of him, but I don't like his […] so.

Interviewer: [But] you live in a safe, good relationship today?

Magdalena: Yes, I do.

Interviewer: It works well and so?

Magdalena: Absolutely. And we have a lot of mutual respect for each other and we strengthen each other, all the time, we make sure to call each other every day and we always tell each other that we love one another and that we always are there [for each other] and we do that little extra. And we both do it and we do it for the children. It's exactly the kind of life you want, and it has helped too, through a lot.

Mutual respect, love (or kindness), and affirmation were described as important factors in a relationship, which in the quotation are contrasted with fear and communication, described as unpleasant or even scary. That is, the former relations with the ex-partner as well as the father were contrasted with a good present relationship, highlighting its importance for wellbeing.

In addition, relationships were compared to an idea and social norms of good relationships and/or partnerships as shown when acknowledging that their life choices and relationships may not look like or work in the same way as other people's relationships with friends and family:

I would say that the balance between us may not be as a normal one, that I’m responsible for shopping grocery and doing the dishes and taking care of the everyday things, because I still have challenges with it. But I have accepted that, since we have a balance in that he takes those chores and I do other things. And he, my husband, also understands that it's because of my history. So instead of feeling that I’m not good enough, I feel that it's okay that I have those challenges and he feels good about taking care of me, so then I allow him to do that too. So, we have found a slightly different function. (Therese)

Therese's narrative shows the importance of a relationship as well as the challenges related to the childhood trauma that affect her relationships and day-to-day life. The narrative shows how past experiences affect the possibility of doing home chores, creating a need for support that can be understood to transgress traditional gender roles or an idea that a relationship is based on mutual responsibility. At the same time, the narrative highlights the importance of a respectful and loving relationship as well as the importance of being understood and supported with respect to wellbeing. Another example of how childhood trauma affects wellbeing with regards to relationships is found in the quotation below:

It has a lot to do with the fact that I have actually accepted my life. I have come to the conclusion that: “Yes, no, I will probably not live in a relationship, because it takes too much of me.” So, I’m not living in a relationship. And somehow, I have chosen not to have children and I’m okay with that. So, that's how I feel. It was really hard for a while that I didn't have children, and I thought that I should have had children, but I feel comfortable with that today. And it has a lot to do with that I have accepted my life, accepted my conditions. (Anna)

To be in a relationship or to have children was most often described in positive terms and as crucial for wellbeing. However, relationships were also described with caution or reservation and, like Anna's narrative, social hardship and choices made as adults were related to childhood maltreatment. To abstain from relationships, whether children, partners, friends, or family members, was described as important for wellbeing even if social norms and expectations could be hard to handle.

In summary, various forms of relationships were given great importance in the women's narratives, especially their relationships with children and partners but also with friends, colleagues, pets, and other family members. They described relationships with a partner, friend, or pet as having a positive effect on their wellbeing in terms of support, respect, trust, understanding, and love. That is, these relationships were related simultaneously to normative ideas about what a good relationship is and in contrast to previous bad relationships such as the relationships they experienced as children.

### Emotional Dimensions of Wellbeing Related to Norms of Happiness

The third dimension of wellbeing relates to feelings and emotions. The women described wellbeing as being free from shame, anger, and fear and maintaining a sense of harmony, control, hope, and safety. Wellbeing was also described as finding a balance in life or a feeling of contentment both in regard to one's self and one's childhood experiences:

Katja: Calm and to be happy with what you have, not wanting things to be different so much. Yes.

Interviewer: Should I interpret it as acceptance or as something else?

Katja: To be satisfied. Acceptance sounds like there are bad things that you are fine with, and that is not really what I mean. More just to be happy.

Interviewer: Be happy and satisfied. That soun[ds like] […] Baloo.

Together: [laugh]

Katja: Yes.

Interviewer: Yes, but like this, be happy. […] Or is it rather the ability to appreciate what is good perhaps?

Katja: Yes, and see it then. Exactly, and don't focus on negative things, or maybe not let negative things take up too much time.

Katja's narrative illustrates how wellbeing was described in terms of feeling happy and content as well as the ability to focus on and appreciate the good things in life. However, in the narratives, the wellbeing experienced today was often contrasted with what life looked like in the past. It was also described as being linked to the present in a way that was particularly related to childhood maltreatment:

Interviewer: Can you say something about your wellbeing?

Elisabeth: Yes. For example, I couldn't appreciate love. I didn't know what love was. And I couldn't trust people. I couldn't recognize people's goodwill because I thought it would repel later. So that's the difference, today I can appreciate love, I can see love. But I’m still afraid of being left. That part is still hard to overcome. Above all, I’m very afraid of […] when I have a partner, I’m afraid that they will just disappear from my life. That's still very strong. But that's, I allow myself to feel loved. I couldn't do that before. […] Thus, that's probably my way of being well. That, I don't constantly have to be afraid of […] yes, but, for my life, quite simply, really.

One of the consequences described by Elisabeth was the feeling of fear in contrast to feeling safe or loved. Positive feelings of today were thus contrasted with negative feelings of the past, that is, related to experiences of maltreatment. In addition, the difference between now and then was also discussed to give perspective on what's important in life today:

I have always found harmony somewhere. I have had my vents. I have had my sanctuary at the island, I have had my painting, I have had my music. I have had all these places where I go, and I feel […] I’ll come to a place where there is a certain grass or […] these little things you can find. […] I have picked up on the small things that make me happy, or the things that have been very hard. But I can say that I have never been afraid of feelings. Once I have felt something, I have been in that moment. And I have felt it, allowed myself to feel it. (Stina)

As illustrated above, childhood experiences allowed for looking at life with gratitude, perhaps in a different way than for those without the experiences of childhood maltreatment. In other words, the childhood experiences of maltreatment gave perspective to what's important in life and, as for Stina, made it possible to feel and appreciate life in a profound way. This included nature, music, and art or, as highlighted by the other women, a day with the kids at an amusement park, a walk with the dog, a hike in the forest, or a cup of favorite coffee. Not to forget, often their experiences of maltreatment were described as affecting their lives in a negative way still today, which included feelings of fear, anxiety, and loss of control, among others, forcing them to actively work with their emotions to allow them to feel safe, loved, or in control, so they could experience wellbeing. Therefore, their past experiences seemed to help them appreciate satisfaction with their life as it is.

### Physical and Mental Dimensions of Wellbeing Related to Norms of What Health Contains

Finally, wellbeing was described by the women as related to the body and health. The narratives included descriptions of various forms of mental and physical health issues, from chronic pain and anxiety to neuropsychiatric diagnoses such as Attention Deficit Hyperactivity Disorder (ADHD) and Posttraumatic Stress Disorder (PTSD). In addition, the relation with and the right to decide issues related to one's own body was a recurring theme in the narratives:

I have had physiotherapists for a hundred years and they have been very important to me, this with daring to feel the body, because I have been terrified. I have worked with that, that my body holds. I have not believed that earlier, because I’m afraid of everything that […] everything that feels in the body, I have been terrified of. I learned to stand and walk and trust my own body. (Anna)

Good health and a good relation to one's body, as shown above, was described as important. In the descriptions of body and health in relation to wellbeing and day-to-day life, a problem or deviation was often pointed out—that is, the body and/or health did not always work according to conceptions of good health or an abled body:

I think it is difficult to have experienced a trauma and not be marked; you are marked, but you live with your wounds in different ways. And I think I was good at keeping those “broken legs” together and to keep on struggling and that made me able to become very well-functioning anyway. (Diana)

The childhood trauma of abuse and neglect, as for Diana, were often related to health issues that affected and limited their lives in different ways in the past. Furthermore, as Diana highlighted, it was explained how living with wounds from childhood affected their adult life, and for some their body and health were described as problematic or as a limitation that still affected every day of their life:

I would say that for the most part I feel very good, satisfied with life, curious about life and trying new things and to meet new people, at the same time my experiences have affected me as a person. I still have some difficulties; I have a pretty strong social phobia that I struggle with all the time, but which is also a bit of an incentive, and it works since if I give in to it, it gets worse, so I have to constantly challenge it, to dare to go outside the door every day, make contact with people and talk and be vulnerable, which is very scary. But I have also learned that I’m very good at hiding it, which means that […] it also gives me self-confidence, that if others don't see it, that I have anxiety, then it doesn't necessarily have to take up so much space. So, it is these two sides, that I control my own life and my wellbeing very much, but at the same time I have a lot of things that I struggle with. (Petra)

As shown, pain or periods of depression or experiencing the consequences of anxiety or phobias were described as related to their trauma and still affecting day-to-day life. However, despite this, the women described their bodies and health as sufficient and able, and, like Petra, they said that they feel well, content, and confident and that they are able to have a functioning adult life, including having a job and good relationships despite various problems with their mental and/or physical health. Thus, the dimensions that constitute wellbeing in the women's descriptions were interconnected.

## Discussion

Based on the importance of understanding how individuals with potentially life-changing or life-shaping experiences describe wellbeing, we set out to explore and analyze how adult women in Sweden exposed to childhood maltreatment describe wellbeing. Our findings support the rendering wellbeing as having multiple aspects (Kahneman et al., 2010) and also reveal that wellbeing, at least among women maltreated as children, is relative. Wellbeing is relative in the sense that it is described and (re)negotiated in relation to both social norms, concerning wellbeing and what it means to be a well-functioning adult and citizen, and to their childhood experiences of maltreatment. Furthermore, our study supports previous findings revealing that wellbeing as a subjective phenomenon embraces how individuals appraise their lives, the events they experience, their physical and mental health, and their living conditions in relation to their own standards (cf. Diener, 2006; Diener & Suh, 1997). Our study expands this knowledge as we found that these aspects are relevant also to the specific group of women maltreated as children and that, for these women, a central aspect of wellbeing is that it is relative. The concept of relative wellbeing highlights dimensions not commonly acknowledged in research about subjective wellbeing. We believe that developing relative wellbeing as a theoretical concept could enable the identification of other points of reference that other groups use to define and describe wellbeing or the way wellbeing is (re)negotiated.

### Wellbeing Relative to Social Norms

Social norms are the informal rules that guide the behavior of individuals and groups by including thoughts about what others do or will do as well as beliefs about what others think one ought to do (Bicchieri, 2006). However, social norms are also formed by individuals’ behaviors in their interactions. Therefore, although individuals risk being sanctioned for going against social norms in the form of internal guilt, shame, gossip, or mocking from acquaintances (Bicchieri & Muldoon, 2011), challenging norms can be an attempt to renegotiate, change, and expand such norms. The multiple aspects embedded in the conceptualization of wellbeing include objective indicators such as employment, housing, education, and having children (Diener & Suh, 1997; Kahneman et al., 2010; Western & Tomaszewski, 2016) as well as the individual's evaluation of and satisfaction with these life circumstances (subjective wellbeing) (Diener, 2006; Kahneman et al., 2010). Furthermore, subjective wellbeing includes the dimension of happiness in terms of enjoyable activities, positive mood, and good health (Kahneman et al., 2010; Krueger et al., 2009). Therefore, not having a job, education, children, or good health can be understood as deviating from a normative understanding of wellbeing. Our findings show how such normative understanding is challenged by describing and renegotiating wellbeing as relative to notions of wealth, health, and happiness. Such renegotiations can be considered as attempts to change and expand existing norms. Findings revealing experiences of wellbeing, for example, not working full-time, not having children, medicating, and/or having health problems, force such non-normative experiences to be included in understandings of subjective wellbeing.

Another finding that contributes to the field of knowledge on women's wellbeing regards possible cultural differences and builds further on Kahneman et al.’s (2010) finding that the basic structure of wellbeing is the same for women in the United States and France, but the specific sources from which they draw happiness is slightly different, reflecting differing norms and social arrangements. We did not find any research from other cultural settings that examined the role of nature and pets as sources of wellbeing. As Capaldi et al. (2014) show, nature can contribute to one's subjective wellbeing, but it differs with childhood experiences and culture. Whether the role of nature and the importance of pets reflect that the sample in our study consists of Swedish people or of women maltreated in their childhood needs further investigation. On one hand, Ahmadi and Ahmadi (2015) found that among cancer patients in Sweden, nature was the most important coping method andan important resource for dealing with the illness, including listening to “natural music” (birds singing and the wind), walking, or engaging in activity outdoors. Similarly, Herlitz points out that “Swedes generally speaking have an almost sacred relationship to nature” (1995, p. 36). On the other hand, reviews find that nature-based therapies in other cultural contexts have a positive impact on the experience of symptoms, quality of life, and self-efficacy among individuals who have mental health issues, such as PTSD, anxiety, or depression (e.g., Möller et al., 2018).

Accordingly, wellbeing is not only described in relation to the four interconnected dimensions—Material and/or economic; Social and relational; Emotional; and Physical and/or mental—but are also renegotiated and described as relative to social norms related to each dimension and what it means to have a well-functioning adult life. The women's narratives can thus be said to widen the perspective on wellbeing, including norms regarding health, social relations, gender roles, and what it means to be a well-functioning adult.

### Wellbeing Relative to Personal Past Experiences

Our study reveals, as mentioned above, that for the participating women wellbeing was relative. It was described, however, not only in relation to social norms but also in comparison with worse situations, including situations not part of their childhood maltreatment. Janoff-Bulman (1992) highlights that individuals often find new meaning and self-worth after experiencing traumatic events and that they come to terms with bad experiences by comparing their experiences with others or hypothetical worse situations or by perceiving themselves as strong and capable enough to deal with an extreme situation. Rather than comparing their own situation with others, our findings reveal that the relativeness of wellbeing comprises comparisons with previous experiences related to trauma, including violence, abuse, and neglect. When compared with earlier experiences, current emotional state and living conditions were described as more benevolent than earlier experiences. Therefore, for this particular group of individuals, it might be that the understanding of wellbeing as an adult rests on the comparison with deficient wellbeing or absence of wellbeing earlier in life because of childhood maltreatment.

Experiencing wellbeing despite childhood maltreatment, as posed by our findings, is a feature of positive adaptation. Positive adaptation (or resilience) after maltreatment has been studied for decades (e.g., Rutter, 2012; Werner, 2013), but mainly with a focus on experiences and conditions in childhood (Liebenberg & Moore, 2018). The women in our study describe such positive adaptation in terms of subjective wellbeing despite their childhood maltreatment. The relativeness in the descriptions of wellbeing posed by our findings corresponds with the understanding of resilience as a dynamic process including interactions among individuals and the environment (Gunnarsdóttir et al., 2021). The women in our study described how wellbeing is multi dimensional and something they had achieved during their life course rather than something they always had possessed. That is, wellbeing can be achieved through resilience. Further research is needed to understand how the process of resilience can be promoted.

In conclusion, wellbeing is described as relative, relating both to the women's earlier experiences and to normative understandings of wellbeing. Wellbeing is intimately and reciprocally connected to having a well-functioning adult life, but among maltreated women, wellbeing and well-functioning are mainly related to the women's own appraisal of their emotional state and life situation rather than external criteria of (objective) wellbeing or a well-functioning life.

### Limitations and Suggestions for Future Studies

Like any study, the results must be read bearing in mind its limitations. These include the sample being relatively small and self-selected and the risk that ideas about social desirability may have affected what our respondents described in the interviews. This study had a specific focus on exploring and analyzing how maltreated women describe wellbeing which can be considered both a strength and a limitation. It is a strength since women are exposed to childhood maltreatment, especially sexual abuse, to a larger extent than men and a deeper understanding of their perspective is thus valuable. On the other hand, it can be considered a limitation since the knowledge of men's experiences is also scarce and would be valuable. Thus, in the future, a study addressing the wellbeing of men with experiences of maltreatment in childhood would be very valuable and further knowledge about and understanding of post-traumatic growth. Similar studies on young people who have more recently dealt with childhood maltreatment would also be valuable. In such studies, we suggest testing the viability of relative wellbeing as a theoretical concept for analyzing the experiences of previously maltreated people and developing it according to new findings to more fully understand their experiences. Also, we suggest future study designs that enable comparisons of responses across gender, culture, nature of childhood maltreatment, and other potentially important characteristics. Mixed methods studies could potentially contribute to a more holistic understanding of wellbeing after maltreatment.

### Implications for Policy and Practice

Bearing the limitations noted above in mind, however, the results of our study have several possible implications for policy and practice. It is important that professionals within welfare organizations, such as health care and social services, are aware of the relative and multiple ways that wellbeing can be understood and experienced and that people who were maltreated as children may experience multifaceted wellbeing as adults while their childhood experience continues to affect their lives. Related to this, it is of importance to problematize the norms of health and wellbeing as well as work, family, and social life to acknowledge the various ways previously maltreated women, as well as other groups, appraise their lives and therefore their possibility to organize, for example, work and family life in flexible ways.
